# Analysis of the clinical characteristics and risk factors associated with contralateral hip fracture after initial hip fracture in elderly patients: a retrospective cohort study

**DOI:** 10.1038/s41598-024-65165-3

**Published:** 2024-06-21

**Authors:** Liping Zhao, Shoujin Tian, Weiping Sha, Liming Wang, Youjia Xu

**Affiliations:** 1 Department of Orthopaedics, The Affiliated ZhangjiagangHospital of Soochow University, Suzhou, China; 2 Department of Orthopaedics, The Second Affiliated Hospital of Soochow University/Osteoporosis Institute of Soochow University, Suzhou, China

**Keywords:** Medical research, Risk factors

## Abstract

Fractures of the contralateral hip may easily occur in elderly patients after an initial hip fracture. The aim of this study was to investigate the clinical characteristics and major predisposing risk factors of contralateral hip fracture after initial hip fracture in the elderly, to provide a clinical basis for preventing contralateral hip fracture. The data of 1586 patients who had sustained first or second hip fractures and had been surgically treated in our department were retrospectively analyzed. Potential predictive factors for contralateral hip fracture and descriptive statistics associated with surgery (such as blood loss, operation time, and length of hospital stay) were recorded. Of these patients, 133 (8.4%) suffered contralateral hip fracture. The rates of contralateral fracture after femoral neck and intertrochanteric fracture were 5.4% and 10.7% respectively (P < 0.01). Fifty-four cases of contralateral hip fracture occurred within one year, an incidence of 40.6%, while 95 cases (71.4%) and 105 cases (78.9%) occurred within two and three years, respectively, with a interval duration of 21.6 months. The risk factors for contralateral hip fracture were found to be age, type of first fracture, bone mineral density, the Singh index, and concomitant internal medical diseases, which were found to be significantly associated with an increased risk of contralateral hip fracture in multivariate logistic regression analysis (P < 0.05). In conclusion, the presence of concomitant internal diseases, type of first fracture, bone mineral density, the Singh index, and age were found to be significant predictors of the risk of contralateral hip fracture in elderly patients after a first hip fracture.

## Introduction

The overall aging of the world's population has led to a gradual increase in the incidence of hip fractures, including intertrochanteric and femoral neck fractures, which is recognized as a major public health problem in many regions of the world. Contralateral hip fracture often occurs in elderly patients following an initial hip fracture. Omsland et al. reported that 11% of males and 15% of females will experience a contralateral hip fracture within 10 years of a first hip fracture^1^. The incidence of the contralateral hip fracture is estimated to be between 6.8% and 16% in elderly patients after a first hip fracture^2,3^, with mortality rates of 15.9% within one year of an initial hip fracture and 37.3% within one year of a contralateral hip fracture^4,5^. Elderly patients with a second hip fracture frequently show reduced mobility, less social independence, and higher rates of mortality, as well as higher rates of dementia^6^. Contralateral hip fractures place a heavy burden on both families and society. It is thus important to analyze and identify the relevant risk factors and clinical characteristics associated with contralateral hip fracture.

The primary goal of this study was to analyze the clinical characteristics and predictors of contralateral hip fracture in elderly patients following an initial hip fracture, thus providing clinical data for the effective prevention of contralateral hip and osteoporotic fractures.

## Results

A total of 1586 patients with hip fracture underwent surgery at our clinical center, including 916 females and 670 males. The overall mean (standard deviation, SD) age at the first fracture was 78.4 (3.3) years and that for contralateral fracture was 81.3 (4.7) years (Table 1). Women (mean [SD] = 83.7 [4.6] tended to be significantly older than men (mean [SD] = 77.9 [10.1] years; p < 0.001). There were statistically significant differences in age, primary fracture type, BMD, the Singh index, and concomitant internal medical diseases between patients who experienced only one fracture (group 2) and those who experienced both initial and contralateral fractures (group 1) (P < 0.05), but no significant differences were seen in terms of other indices (P > 0.05), including Harris score for hip function. The clinical data are summarized in Table 1. A total of 133 patients (57 males and 76 females; 8.4%) suffered a contralateral hip fracture (Table 1), indicating an absolute risk of 15.7% (Table 2). In the first, second, and third years following the initial fracture, the risk of contralateral hip fracture was 2, 1, and 1%, respectively. The median interval time between the two hip fractures in all 133 patients was 21 months (IQR 13.0, 25.0). Contralateral fractures occurred in 40.6% and 71.4% of cases within 1 and 2 years, respectively, with 78.9% occurring after 3 years (Table 2), indicating that the frequency of contralateral hip fractures increases over time.

**Table 1:** Demographic and clinical characteristics of the two groups.

Factor	Total patients, n = 1586	Group 1, n = 133	Group 2, n = 1453	Test statistics	P value
Age (years)	80.2 ± 6.4 (range 68 to 95)	81.3 ± 4.7 (range 68 to 95)	78.4 ± 3.3 (range 60 to 92)	16.980	< 0.001*
60 ~ 70 (years)	469 (29.5%)	12 (9.0%)	457 (31.5%)		
71 ~ 80 (years)	529 (33.4%)	49 (36.8%)	480 (33.0%)		
81 ~ 90 (years)	464 (29.3%)	56 (42.1%)	408 (28.1%)		
≥ 91 (years)	124 (7.8%)	16 (12.1%)	108 (7.4%)		
Sex				0.297	0.895
Male	670 (42.2%)	57 (42.9%)	613 (42.2%)		
Woman	916 (57.8%)	76 (57.1%)	840 (57.8%)		
First fracture type				16.102	0.022*
Femoral neck fracture	648 (40.8%)	39 (29.3%)	673 (46.3%)		
Intertrochanteric fracture	938 (59.2%)	94 (70.7%)	780 (53.7%)		
Osteoporosis(BMD)				12.105	0.001*
Yes	1214 (76.5%)	122(91.7%)	1092 (75.2%)		
No	372 (23.5%)	11(8.3%)	361 (24.8%)		
Osteoporosis medication				3.048	0.416
Yes	905 (57%)	90 (67.6%)	815 (56.1%)		
No	681 (43%)	43 (32.4%)	638 (43.9%)		
Singh index grading				14.172	0.001*
Grade 1 ~ 3	1091 (68.7%)	116 (87.2%)	975 (67.2%)		
Grade 4 ~ 6	485 (31.3%)	17 (12.8%)	478 (32.8%)		
Concomitant internal medical diseases				11.663	0.000*
Yes	932 (58.8%)	99 (74.4%)	833 (57.3%)		
No	654 (41.2%)	34 (25.6%)	620 (42.7%)		
Time from injury to surgery(h)	50.4 ± 3.1 (range 12 to 120)	52.6 ± 1.6 (range 13 to 120)	49.3 ± 1.1 (range 12 to 96)	1.479	0.140
Type of implant				2.538	0.319
AFHR	314 (19.8%)	14 (10.5%)	300 (20.6%)		
THR	353 (22.2%)	25 (18.8%)	328 (22.6%)		
Cannulated screw	45 (2.8%)	0 (0%)	45 (3.1%)		
Locking plate	87 (5.5%)	0 (0%)	87 (6.0%)		
PFNA	668 (42.2%)	88 (66.2%)	580 (39.8%)		
DHS	119 (7.5%)	6 (4.5%)	113 (7.8%)		
Operation time (min)	56.1 ± 4.7 (range 35 to 140)	54.7 ± 1.4 (range 35 to 90)	55.1 ± 3.1 (range 40 to 140)	4.191	0.264
Length of hospital stay (days)	7.6 ± 1.7 (range 3 to 15)	7.8 ± 2.8 (range 4 to 15)	7.3 ± 2.7 (range 3 to 14)	7.135	0.183
Blood loss (ml)	240.3 ± 2.8 (range 130 to 380)	235.8 ± 1.9 (range 130 to 300)	241.4 ± 3.8 (range 150 to 380)	1.461	0.492
Harris score (x ± s)	86.6 ± 3.1 (range 80 to 98)	83.7 ± 7.0 (range 80 to 95)	88.1 ± 7.6 (range 84 to 98)	1.200	0.231
Concomitant internal medical diseases				χ^2^ value	P value
Cataract	155 (9.8%)	26 (19.5%)	129 (8.9%)	4.514	0.037
Cerebrovascular disease	369 (23.3%)	52 (39.1%)	317 (21.8%)	5.196	0.033
Neurological diseases	352 (22.2%)	59 (44.4%)	293 (20.2%)	5.293	0.021
Rheumatoid Arthritis	159 (10.0%)	23 (17.2%)	136 (9.3%)	4.766	0.043
Essential Hypertension	621 (39.2%)	53 (39.8%)	568 (39.1%)	5.026	0.087
Diabetes	303 (19.1%)	42 (31.6%)	261 (17.9%)	5.238	0.028
Lung disease	274 (17.3%)	25 (18.7%)	249 (17.1%)	5.147	0.064

AFHR indicates artificial femoral head replacement.

*THR* total hip replacement, *PFNA* proximal femoral nail anti-rotation; DHS:dynamic hip screw.

*Asterisks represent significant differences.

**Table 2:** The incidence, risk per year, and median interval time of contralateral hip fractures.

	1 year	2 year	3 year	Absolute risk
Contralateral hip fracture incidence	54 (40.6%)	95 (71.4%)	105 (78.9%)	/
Contralateral hip fracture risk	2%	1%	1%	15.7%
Interval time (months), median(IQR)	21.0 (13.0, 25.0)			

Annotation: The frequency of contralateral hip fractures increases over time. Risk refers to the probability of an event (such as a fracture) occurring within a certain period of time. Incidence refers to the ratio of the number of occurrences of a certain event within a certain period of time to the overall number of related events; *IQR* Inter quartile range.

In terms of comorbidities, the proportions of cataracts (0.037), cerebrovascular disease (0.033), rheumatoid arthritis (0.043), neurological diseases (0.021), and diabetes (0.028) in group 1 were significantly higher than those in group 2 (P < 0.05, Table 1). Lower Singh indices and bone mineral density (BMD) were associated with aging, as seen in the age groups presented in Table 3. There was a significant association between the incidence of contralateral hip fracture and lower Singh index (grade 3 or lower) and BMD (p < 0.001, Table 4). Patients with lower Singh indices and BMD values aged 71–80 years, 81–90 years, and above 91 years had 3.41-,7.38-, and 11.19-fold greater chances, respectively, of experiencing a contralateral hip fracture (Table 3). The incidence of contralateral hip fracture after intertrochanteric fracture was higher than that of femoral neck fracture (P = 0.001), and was associated with both older age and more severe osteoporosis the age and osteoporosis than that of femoral neck fracture (P < 0.05, Table 4).

**Table 3:** Relationship between age, Singh index grading, and BMD.

Age (years)	Singh index		BMD(T value)			OR (95% CI)	P-value
	Grade1 ~ 3 (n = 710)	Grade4 ~ 6 (n = 876)	− 2.5 ~ − 3.0 (n = 514)	− 3.0 ~ − 3.5 (n = 364)	<-3.5 (n = 336)		
	N (%)	N (%)	N (%)	N (%)	N (%)		
60 ~ 70	107 (22.8)	362 (77.2)	149 (31.7)	72 (15.3)	26 (5.5)	1.17 (0.93–4.18)	0.041
71 ~ 80	217 (41.0)	312 (59.0)	223 (42.1)	116 (21.9)	41 (7.5)	3.41 (3.31–12.6)	0.026
81 ~ 90	271 (58.4)	193 (41.6)	114 (24.5)	178 (38.3)	172 (37.0)	7.38 (5.68–20.9)	0.001
≥ 91	115 (92.7)	9 (7.3)	7 (5.6)	19 (15.2)	99 (79.2)	11.19 (6.54–25.1)	0.001

% The percentage sign represents the proportion.

**Table 4:** The relationship between contralateral fractures, osteoporosis, and the incidence of contralateral fracture types.

Contralateral fracture types	Cases(n)	Incidence	Average age (y)	Osteoporosis	
				BMD	Singh index
Femoral neck fracture	1453	39(5.8%)	77.6 ± 2.14	− 3.475 ± 0.33	86.37 ± 7.38
Intertrochanteric fracture		94(12.1%)	84.2 ± 3.71	− 3.317 ± 0.19	86.45 ± 6.93
P value		0.001*	0.026	0.038	0.022

*Asterisks represent significant differences.

The OR values of these factors shown in Table 5 are greater than 1 with P-values less than 0.05, indicating that concomitant internal medical diseases, osteoporosis (BMD and Singh index), age, and type of first fracture were key risk factors for developing contralateral hip fracture in elderly patients, as shown by multivariate logistic regression analysis. Lower Singh index (OR = 21.512;95%CI = 2.397,17.517; P = 0.001) and BMD (OR = 26.793;95%CI = 2.426,24.741; P = 0.001) had the strongest correlations with the occurrence of contralateral fracture, with patients whose Singh index was grade1-3 and BMD(T<-3.0) or lower at the first hip fracture having 21.5-fold and 26.7-fold increased risks, respectively, for sustaining contralateral hip fractures (Table 5). Concomitant internal medical diseases (OR = 6.155; 95%CI = 1.967, 15.212; P = 0.019), age (OR = 8.502;95%CI = 2.326,11.852; P = 0.012), and the type of initial fracture (OR = 7.173; 95%CI = 2.038,20.724; P = 0.026) were highly associated with an increased risk of sustaining contralateral hip fractures(6.1-, 8.5-, and 7.1-fold respectively)(Table 5).

**Table 5:** Results of multivariable logistic regression analysis of factors affecting contralateral hip fracture.

Factor	Β value	OR value	95% CI	P value
Concomitant internal medical diseases	2.347	6.155	1.967, 15.212	0.019
BMD (T<-3.0)	1.918	26.793	2.426, 24.741	0.001
Age	2.106	8.502	2.326, 11.852	0.012
Singh index(grade1-3)	2.037	21.512	2.397, 17.517	0.001
First fracture type	2.84	7.173	2.038, 20.724	0.026

## Discussion

Hip fractures, including both femoral neck fractures and intertrochanteric fractures, tend to occur more frequently in the elderly population, with some patients having a higher risk for subsequent contralateral hip fracture due to risk factors. Although several studies have elucidated predictors associated with an increased risk of a second hip fracture, such as neurological disease, falls, dizziness, eye disease, and the Singh index^7,8^, the present study investigated which risk factors associated with the initial hip fracture were still operative as risk factors for contralateral hip fracture in the elderly, which would be useful for the prevention of such fractures.

The incidence of contralateral hip fracture in the present study was found to be 8.4%, which is approximately consistent with those reported in previous studies (range, 4.3‒16.1%)^1,9,10^. The difference in incidence between these studies may be related to the ages of the enrolled patients^11^ and follow-up times^3^. In the present study, all patients were over 60 years old and were followed up for 8 years. In addition, patients with old fractures were excluded, which may partially account for the difference. It was previously reported that 21.4% and 78.6% of second hip fractures occurred within and beyond 12 months, respectively, after the first hip fracture, respectively^7^. Here, it was found that 40.6% of the contralateral hip fractures occurred within a year, 71.4% within two years, and 78.9% after three years, with a median interval of 21 months, which was longer than the interval reported by Angthong et al.^7^. The reason could be due to a lack of long-term and systematic rehabilitation training following discharge. Patients with poor hip function after the first hip surgery, combined with lower limb muscle atrophy resulting from being bedridden for long periods, leading to poor muscle strength and ability to balance, as well as the coordination and function of the hip joint, were more prone to sustain contralateral hip fracture with a fall, which may be associated with a higher tendency for contralateral hip fracture occurrence within three years. Research has shown that postoperative muscle weakness around the hip is an important contributor to contralateral fracture after initial hip fracture surgery^12^, and early postoperative rehabilitation training can enhance hip muscle strength and improve hip joint mobility^13^. Therefore, improving both muscle strength and joint movement after initial hip-fracture surgery would be beneficial in preventing contralateral fracture.

The present data revealed that the incidence of contralateral fractures being intertrochanteric fractures (12.1%) was higher than that of being femoral neck fractures (5.8%). The reason could be that patients with intertrochanteric fractures were older and had more severe osteoporosis, together with a history of fracture, compared with those with femoral neck fractures^14^. Furthermore, the mean age and degree of osteoporosis were greater at the time of contralateral fracture occurrence, indicating that the femoral intertrochanteric region was more susceptible to contralateral fracture when the hip was subjected to minor external force^8^.

It is well-known that osteoporosis and bone fragility worsen with age, which unquestionably increases the risk of hip fracture due to bone brittleness. Clinical reports on the effect of age on the incidence of contralateral fracture tend to be inconsistent^15,16^. Age has been regarded as an influencing factor^17^, and under normal conditions, it cannot lead directly to contralateral hip fracture, which occurs only when low-energy injuries are sustained. However, advanced age was indeed found to be the main risk factor associated with contralateral hip fracture in the elderly, just as older age is typically linked to a higher risk of secondary osteoporotic fracture^17,18^. The present findings also revealed a firm correlation between lower Singh index, BMD, and age over 81 years during which mobility and bone mass were significantly reduced. Furthermore, the Singh index and BMD typically decline substantially with aging, which may reflect the relationship between osteoporosis, age, and contralateral hip fracture occurrence. Therefore, osteoporosis plays a key role in the occurrence of contralateral fracture, and targeted anti-osteoporosis therapy can prevent further bone mass loss, effectively preventing contralateral fracture^10^.

Cataracts, cerebrovascular disease, neurological diseases, rheumatoid arthritis, and diabetes were defined as major risk factors in the univariate analysis of concomitant internal medical diseases associated with contralateral hip fracture. It is likely that diseases such as cataracts, cerebrovascular disease, and neurological disease can impair an elderly patient's ambulatory ability and body balance due to impaired postural control or cognitive impairment, making them more prone to falling. However some diseases, such as diabetes and rheumatoid arthritis, may be associated with osteoporosis due to long-term medication with drugs that reduce bone strength and increase bone fragility, thereby markedly increasing the risk of contralateral fracture. Hence, concomitant internal medical diseases were clearly associated with subsequent contralateral fractures. Given that patents with their first hip fracture and untreated concomitant internal medical diseases were found to be at a high risk of controlateral hip fracture, it is recommended that active intervention and treatment of internal medical diseases be initiated early.

The key to preventing contralateral hip fracture is to identify and control the relevant predictive risk factors. The results showed that BMD, Singh index, concomitant internal medical diseases, type of first fracture, and age were the most significant risk factors for contralateral hip fracture, with BMD, the Singh index, and age being the most dangerous, which is partially consistent with previous studies^19,20^, and that both lower Singh index and BMD were positively correlated with the risk of contralateral hip fracture. We discovered that senior patients with osteoporosis, basic internal medicine diseases, and a first hip fracture, were more likely to sustain a contralateral hip fracture. Clarifying the role of prognostic factors associated with the development of a contralateral fracture is critical for the identification of high-risk groups and the development of preventive interventions. Elderly patients with an initial hip fracture should avoid reinjury within three years, and concomitant internal medical diseases should be actively treated, together with treatment of osteoporosis and the maintenance of muscle strength and balance, all of which can effectively reduce the risk of contralateral hip fracture. Neither age nor type of initial fracture can be changed, but we may prevent falls and hip fractures by vigorous exercise and muscle strengthening.

The advantage of the study was that it provides data that could be beneficial in the identification of risk factors that can be treated and corrected to reduce the frequency of contralateral fractures within three years. However, the study has several limitations. First, while multiple factors can contribute to contralateral fractures, only some of these were addressed in this study, and further assessment of other potential factors is required for the objective and comprehensive evaluation of the risk factors for contralateral fractures. Second, the study was a single-center investigation, which may limit the generalizability of the findings.

## Methods

### Statement

This is a retrospective clinical study with no extra involvement of patients. The research ethics committee of our hospital research ethics committee confirmed that no ethical approval was required. The research was performed in accordance with the Declaration of Helsinki and relevant guidelines and regulations (Supplementary information).

### General information on patients

This retrospective study included 1586 patients with hip fractures who were surgically treated at our trauma center between 2008 and 2017. The inclusion criteria were: (1) Age ≥ 60 years; (2) Fracture caused by low-energy injury. The exclusion criteria were:(1) Conservative treatment; (2) Simultaneous bilateral simultaneous hip fractures;(3) Fractures resulting from pathological fractures;(4) Old fractures; (5) Patients with osteoporosis who were taking anti-osteoporosis medication such as bisphosphonates. Paper charts and the hospital's electronic database (Lis) were used to collect the study variables. All variable data of the hip fractures were scored. Patients were divided into group 1 (n = 133, patients with contralateral hip fracture) and group 2 (n = 1453, patients without contralateral hip fracture).

The variables assessed were sex, age, type of first fracture, concomitant internal medical diseases, BMD, the interval between fractures, hip Harris score (last follow-up), operation time, type of treatment (i.e., type of implant), blood loss, days spent in hospital, osteoporosis medication, and the Singh index. The BMD was measured by digital dual-energy X-ray absorptiometry and corresponded to the diagnostic criteria specified by the World Health Organization (WHO). Concomitant internal medical diseases included rheumatoid arthritis, essential hypertension, diabetes, neurological diseases, cataracts, cerebrovascular diseases, and lung diseases. The X-rays (hip and pelvis) taken at the initial hip fracture were examined to assess the degree of osteoporosis. Trabecular changes in the proximal femoral trabeculae were observed on the anteroposterior radiographs of the contralateral unfractured hip according to the Singh index criteria^21^. Trabecular changes were graded separately by two experienced senior orthopedists. The hip function was assessed by the hip Harris score standard^22^. Surgery was performed by several senior surgeons who specialized in trauma.

### Statistical analysis

Statistical analysis was performed using SPSS version 22.0. Continuous variables are presented as the mean and standard deviation, and categorical variables are presented as percentages. Chi-square tests were used for comparing groups of categorical data, while independent t-tests were used to compare groups of continuous data. P<0.05 was considered to be statistically significant. Multivariable logistic regression analysis was performed with variables showing p<0.05. The age-corrected probability rate (PR) and 95% confidence interval (CI) were set using the maximum probability method. Subgroup analysis was performed with variables that were found to be statistically significant in the logistic regression analysis to investigate the major risk factors for contralateral hip fracture.

### Ethics approval and consent to participate

All included patients gave their oral and written informed consent. The study was approved by the Ethics Committee of Zhangjiagang Hospital Affiliated to Soochow University (approval no.ZJGYYLL 2022 08 lw001).

### Supplementary Information

Supplementary Information.

## Data Availability

All data are available within the Article and Supplementary Files or available from the corresponding authors upon reasonable request.
